# Omental arteriovenous fistula after splenectomy treated with transarterial embolization

**DOI:** 10.1186/s42155-023-00374-x

**Published:** 2023-04-26

**Authors:** Takayuki Sanomura, Takashi Norikane, Satoshi Uchinomura, Yasukage Takami, Toshiya Ensako, Mina Nagao, Akihiro Deguchi, Keiichi Okano, Yoshihiro Nishiyama

**Affiliations:** 1 Department of Radiology, Faculty of Medicine, Kagawa University, 1750-1 Ikenobe, Miki-Cho, Kita-Gun, Kagawa 761-0793 Japan; 2 Department of Radiology, Kagawa Rousai Hospital, 3-3-1, Joutou, Marugame, Kagawa 763-8502 Japan; 3 Department of Gastroenterological Surgery, Faculty of Medicine, Kagawa University, 1750-1 Ikenobe, Miki-Cho, Kita-Gun, Kagawa 761-0793 Japan; 4 Department of Gastroenterology, Kagawa Rousai Hospital, 3-3-1, Joutou, Marugame, Kagawa 763-8502 Japan

**Keywords:** Omental arteriovenous fistula, Vessel-sealing device, Embolization

## Abstract

**Background:**

Laparoscopic splenectomy for patients with portal hypertension is associated with a high risk of bleeding. The use of vessel-sealing devices and automatic sutures is important for bleeding control. However, a rare complication of abdominal surgery is the direct communication between the arterial and portal circulation related to surgical procedures such as simultaneous ligature of an artery and adjacent vein. We describe a rare case of omental arteriovenous fistula (AVF) after laparoscopic splenectomy treated with transarterial embolization.

**Case presentation:**

We report a case of a 46-year-old male patient with an omental AVF after a laparoscopic splenectomy 6 years ago for splenomegaly associated with alcoholic cirrhosis. Follow-up abdominal dynamic computed tomography accidentally revealed a vascular sac (25 mm in the major axis) that formed an omental AVF with anastomosis to the left colonic vein. The communication was considered to be caused by using a vessel-sealing device. No symptoms related to the AVF were observed. The AVF was embolized with microcoils using the transarterial approach. A 4-axis catheter system was used for accurate embolization due to the long and tortuous distance from the celiac artery. No recurrence or symptoms were observed after 6 months.

**Conclusions:**

Treatment of arterioportal fistula is mandatory, even in asymptomatic patients. Embolization is a less invasive alternative to surgical approaches. The 4-axis catheter system was useful for accurate embolization via a long and tortuous artery.

## Background

Laparoscopic splenectomy for patients with portal hypertension is associated with a high risk of bleeding due to adhesions associated with splenomegaly and the development of collateral vessels (Hashizume et al. 2002). The use of vessel-sealing devices and automatic sutures is important for bleeding control. However, a rare complication of abdominal surgery is the direct communication between the arterial and portal circulation related to surgical procedures (Vauthey et al. 1997). This report describes a case of transarterial embolization of an omental arteriovenous fistula (AVF) with anastomosis to the left colonic vein (LCV), which was considered affected by a vessel-sealing device with simultaneous sealed of the omental artery and vein.

## Case presentation

A 46-year-old male had undergone laparoscopic splenectomy 6 years previously for splenomegaly associated with alcoholic cirrhosis. The follow-up computed tomography (CT) revealed saccular dilatation of the blood vessel from the left gastroepiploic artery (LGA), which was newly diagnosed since the splenectomy. The vascular sac showed an increase in size, but no related symptoms were observed. The patient was referred to our department for embolization. Pre-embolization examination revealed Child–Pugh A (score 6) liver cirrhosis. No other cirrhosis complications such as ascites, variceal hemorrhage, or hepatic encephalopathy were recognized.

**Fig. 1:**
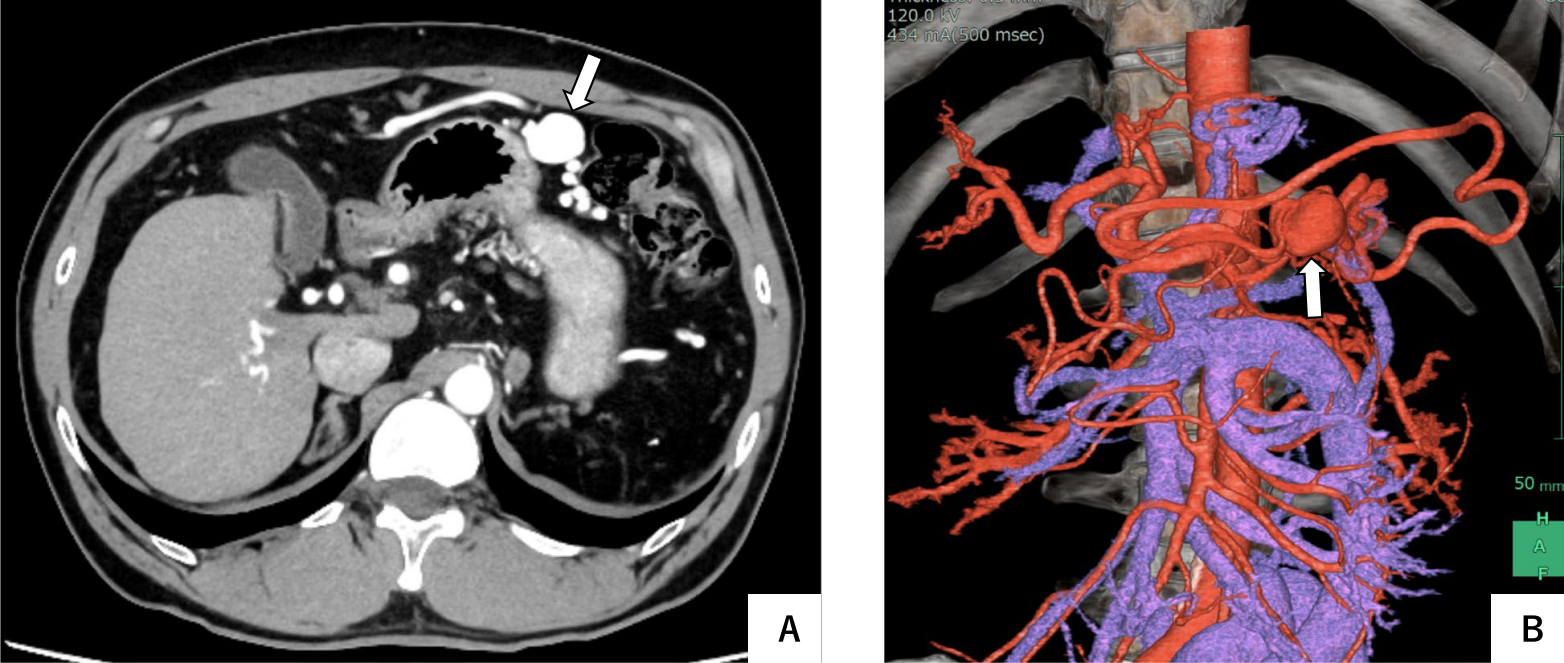
Abdominal contrast-enhanced CT showed an aneurysm-like saccular dilatation in the left upper abdomen (white arrow). **A** Arterial phase; **B** VR image

CT showed a dilated omental artery branching from the LGA that continued to the vascular sac and drained into the omental vein and LCV (Fig. 1). In addition, inferior mesenteric varices associated with portal hypertension were observed, but this had been observed before splenectomy. The vascular sac increased with time and had a major axis of approximately 25 mm, compared to 15 mm in the 4-year-old image. Since it exceeded 20 mm in the major axis, it was determined that treatment was indicated in the same way as for general visceral aneurysms.

**Fig. 2:**
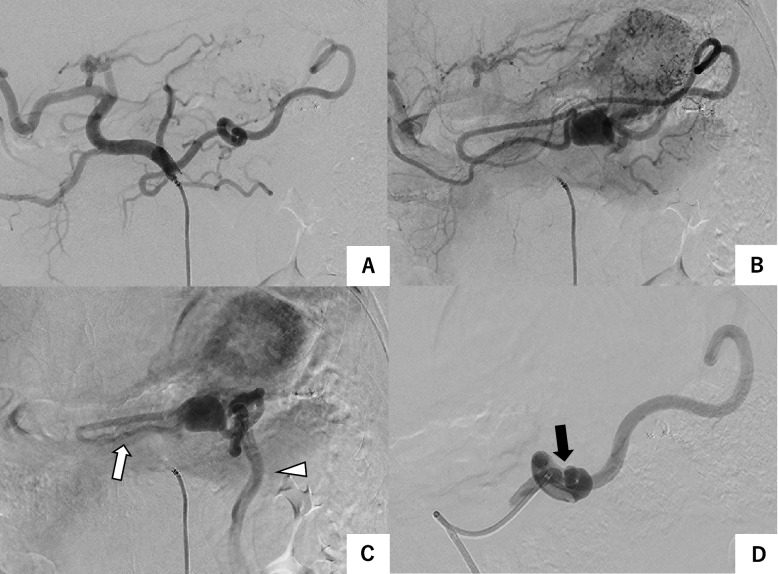
Angiography of the celiac artery showed that the dilated omental artery was revealed continuously from the splenic artery (**A**), turned over, headed toward the vascular sac (**B**), and returned to the omental vein (white arrow) and left colonic vein (white arrowhead) (**C**). A stenosis (black arrow) due to ligation at the time of splenectomy was observed in the splenic artery (**D**)

**Fig. 3:**
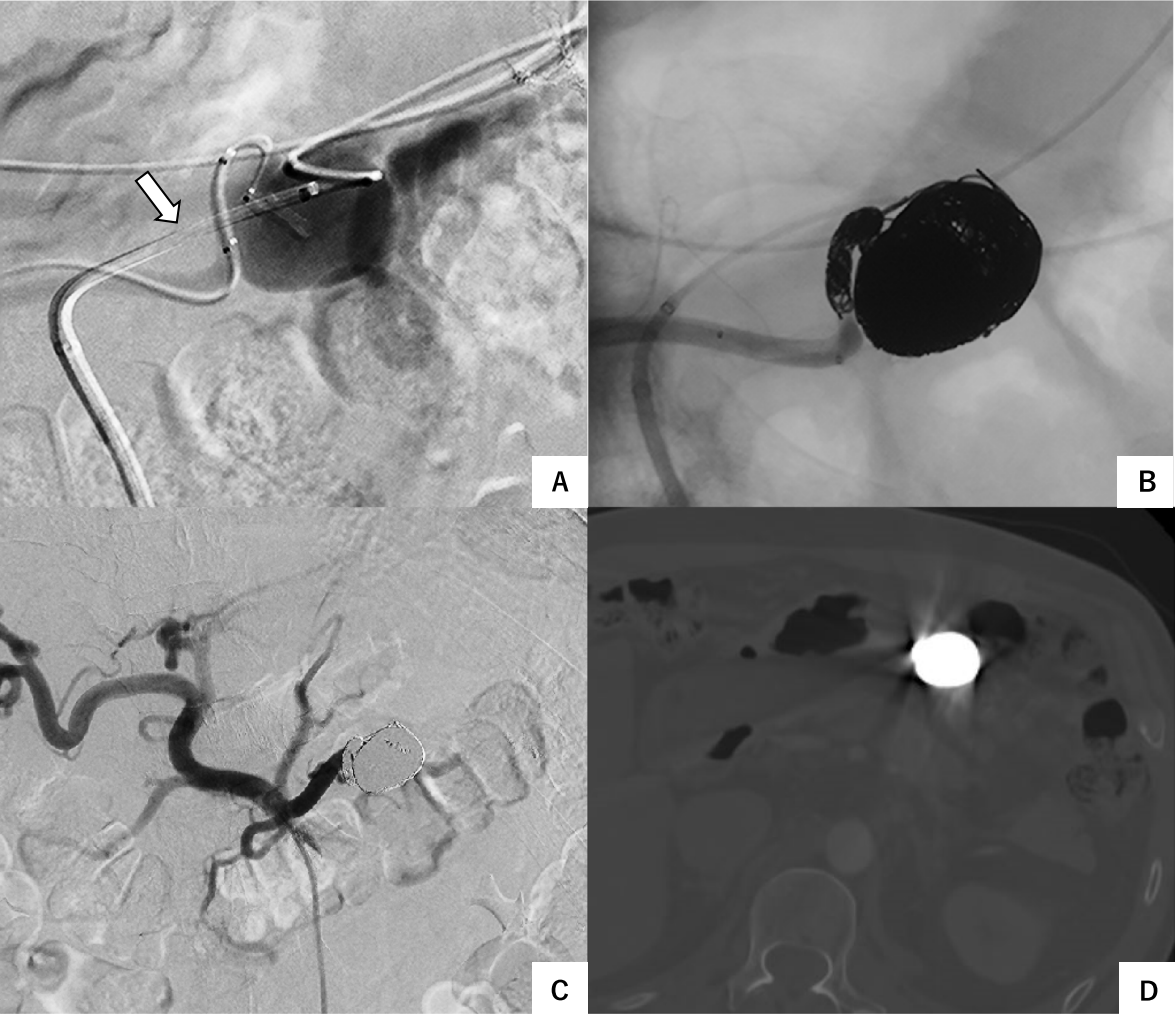
**A** The 4-axis catheter system (white arrow) was used due to the long and tortuous distance from the celiac artery. **B** The sac and the end of the feeding artery were embolized with microcoils. **C** Celiac arteriography after embolization. **D** Contrast-enhanced CT 6 months after embolization showed complete occlusion

A 4.5 Fr guiding sheath (Parent Plus®45; Medikit, Tokyo, Japan) was used to guide the celiac artery. Celiac arteriography also revealed a dilated omental artery of the splenic artery, which made a U-turn at the median level, headed leftward, and drained into the omental vein and the LCV through the sac (Fig. 2). Next, a 6 Fr guide extension catheter (Guidezilla; Boston Scientific, Natick, MA, USA) was used to guide the splenic artery. In the splenic artery, a stenosis that appeared to be a ligation at the time of splenectomy was observed. The omental vein is visualized as folding back into the sac, and at the same time, the left colic vein, which continues from the tortuous vessel, can also be seen. We decided to embolize the sac and the end of the feeding artery. In addition to the guiding sheath and guide extension catheter, a 4-axis system combining a 3.4 Fr/3.2 Fr microcatheter (Guidepost®; Tokai Medical Products, Aichi, Japan) and a 2.6 Fr/2 Fr microcatheter (Excelsior®1018®; Stryker, Fremont, CA, USA) was used due to the long and tortuous distance from the celiac artery (Fig. 3). The sac and the end of the feeding artery were embolized with microcoils. Microcoils with a maximum diameter of 20 mm were placed to fill the sac. A total of 31 microcoils (Target™ [Stryker, Fremont, CA, USA], Avenir® [Wallaby Medical, Shanghai, China], and IDC [Boston Scientific, Marlborough, MA, USA]) were used for embolization. The volume embolization ratio (VER) of the sac was 34.0%. The use of n-butyl-2-cyanoacrylate (NBCA) in combination was also considered, but it was not used considering safety because flow control was not performed. No procedure-related complications were noted. The total procedure time was 210 min and the length of stay of the patient was 4 days. There was no recurrence on CT after 6 months and no complications such as coil migration or portal vein thrombosis were observed.

## Discussion

Arterioportal fistulas (APFs) are rare vascular disorders of the mesenteric circulation. According to Vauthey et al., 16% of APF are due to iatrogenic procedures (Vauthey et al. 1997). Their causes during surgical procedures include direct injury to an artery or vein, mass or transfixion suture ligation of an artery and vein, and infection and necrosis of the vessel wall (Rossi et al. 1974). The time between surgery and diagnosis varied. Defreyne et al. reported an average age of 10 years (Defreyne et al. 2002). APFs appear to develop slowly from a traumatic vascular defect to a wide shunting area with subsequent venous enlargement before becoming symptomatic (Madsen et al. 2008; Bouziane et al. 2011). Traumatic arteriovenous (AV) shunts are usually solitary and involve a single direct communication between the artery and the adjacent vein. The hepatic artery is the most commonly involved, followed by the superior mesenteric and splenic arteries (Vauthey et al. 1997). Splenic AVF has been reported as a complication of splenectomy, and right gastroepiploic AVF after gastrectomy has also been reported (Woźniak et al. 2011; Shigematsu et al. 2008). However, we did not find any reports of omental AVF after splenectomy. Regarding the etiology of this case, an anastomosis between the omental vein and the LCV was already recognized on CT before splenectomy. Communication was considered to occur when the bundle of the omental artery and vein was sealed with a vessel-sealing device.

The treatment of APF is believed to be mandatory even in asymptomatic patients because it prevents the late repercussion of portal hypertension (Bouziane et al. 2011). In addition, the risk of vascular sac rupture was also considered in this case. Surgical excision is highly effective but requires extensive adhesiolysis and exposure of the inflamed mesentery. Endovascular therapy is minimally invasive and the first option of treatment. A covered stent is used if a fistula develops in a large vessel. Embolization techniques are considered appropriate for non-traumatic AV shunts. Cho et al. proposed a classification for AV malformations that have been commonly used for non-traumatic AV shunts (Cho et al. 2006). This case is considered Type 1 of Cho’s classification. The embolization strategy for Type 1 is embolization of the dominant outflow vein or the end of the feeding artery via a transarterial approach. Furthermore, its shape is similar to that of a pulmonary AV malformation. Therefore, we decided to embolize the sac and the end of the feeding artery. The approach artery was thin and tortuous, so it was considered difficult to use a covered stent or a vascular plug. Liquid embolic materials were not used due to the risk of portal vein embolism due to outflow. After the embolization, the patient is under follow-up using CT once every six months to check for recanalization.

## Conclusions

We present a rare case of omental AVF after splenectomy. Treatment of APF is mandatory, even in asymptomatic patients. Embolization is a less invasive alternative to surgical approaches. The 4-axis catheter system was useful for accurate embolization via a long and tortuous artery.

## Data Availability

The data used the study are available from corresponding author on request.

## Abbreviations

AVF: Arteriovenous fistula
LCV: Left colonic vein
CT: Computed tomography
LGA: Left gastroepiploic artery
Fr: French catheter scale
MA: Commonwealth of Massachusetts
CA: State of California
VER: Volume embolization ratio
NBCA: N-butyl-2-cyanoacrylate
APF: Arterioportal fistula
AV: Arteriovenous
